# *Salmonella* and Antimicrobial Resistance in Wild Rodents—True or False Threat?

**DOI:** 10.3390/pathogens9090771

**Published:** 2020-09-21

**Authors:** Magdalena Skarżyńska, Magdalena Zając, Ewelina Kamińska, Arkadiusz Bomba, Jacek Żmudzki, Artur Jabłoński, Dariusz Wasyl

**Affiliations:** 1 Department of Microbiology, National Veterinary Research Institute, 24-100 Puławy, Poland; magdalena.zajac@piwet.pulawy.pl (M.Z.); wasyl@piwet.pulawy.pl (D.W.); 2 Department of Omics Analyses, National Veterinary Research Institute, 24-100 Puławy, Poland; Ewelina.Kaminska@piwet.pulawy.pl (E.K.); arkadiusz.bomba@piwet.pulawy.pl (A.B.); 3 Department of Swine Diseases, National Veterinary Research Institute, 24-100 Puławy, Poland; jaca@piwet.pulawy.pl; 4 Department of Clinic Large Animal Diseases, Faculty of Veterinary Medicine, Warsaw University of Life Sciences, 02-787 Warsaw, Poland; artur_jablonski@sggw.pl

**Keywords:** rodents, AMR, *Salmonella*, genotype/phenotype discrepancy

## Abstract

Transmission of pathogenic and resistant bacteria from wildlife to the bacterial gene pool in nature affects the ecosystem. Hence, we studied intestine content of five wild rodent species: the yellow-necked wood mouse (*Apodemus flavicollis*, *n* = 121), striped field mouse (*Apodemus agrarius*, *n* = 75), common vole (*Microtus arvalis*, *n* = 37), bank vole (*Myodes glareolus*, *n* = 3), and house mouse (*Mus musculus*, *n* = 1) to assess their potential role as an antimicrobial resistance (AMR) and *Salmonella* vector. The methods adopted from official AMR monitoring of slaughtered animals were applied and supplemented with colistin resistance screening. Whole-genome sequencing of obtained bacteria elucidated their epidemiological relationships and zoonotic potential. The study revealed no indications of public health relevance of wild rodents from the sampled area in *Salmonella* spread and their limited role in AMR dissemination. Of 263 recovered *E. coli*, the vast majority was pan-susceptible, and as few as 5 *E. coli* showed any resistance. In four colistin-resistant strains neither the known *mcr* genes nor known mutations in *pmr* genes were found. One of these strains was tetracycline-resistant due to *tet*(B). High diversity of virulence factors (*n* = 43) noted in tested strains including *ibeA*, *cdtB*, *air*, *eilA*, *astA*, *vat*, *pic* reported in clinically relevant types of enteric *E. coli* indicate that rodents may be involved in the ecological cycle of these bacteria. Most of the strains represented unique sequence types and ST10805, ST10806, ST10810, ST10824 were revealed for the first time, showing genomic heterogeneity of the strains. The study broadened the knowledge on phylogenetic diversity and structure of the *E. coli* population in wild rodents.

## 1. Introduction

Increasing antimicrobial resistance (AMR) of bacteria is a challenging problem that poses a threat to public health. AMR led to 33,000 deaths in the European Union (EU) and the European Economic Area (EEA) in 2015 only [1]. Reports from the United States (US) indicate that over 2.8 million antimicrobial-resistant infections occur each year, and more than 35,000 of these cases are mortal [2]. A large fraction of life-threatening infections is caused by *Enterobacterales*. Among them, *Salmonella* constantly remains an important zoonotic hazard noted on a global scale. Salmonellosis was the second most frequently reported zoonosis in humans in the EU/EEA, with 91,857 reported cases in 2018 [3]. In the US, *Salmonella* caused 46.623 infections in 2016 [4]. Shiga toxin-producing *Escherichia (E.) coli* (STEC) with 8161 confirmed cases in the EU and other virulent *E. coli* clones cannot be omitted either [3]. In addition to the pathogens, the role of commensal *Enterobacterales* should be emphasized as they both constitute reservoir and vector of AMR mechanisms and pose a threat, e.g., to people with immunodeficiency.

It is estimated that 60% of human infectious diseases are zoonotic, thus understanding the role of different animal populations in the spread of clinically relevant and resistant bacteria is essential [5]. Animals can be asymptomatic *Salmonella* carriers and may constitute a reservoir of AMR determinants. The role of food-producing animals in the spread of AMR and *Salmonella* along the food chain has been confirmed by numerous monitoring programs [3,6,7,8]. Several reports indicate the contribution of wildlife in AMR and *Salmonella* dissemination [9,10,11]. However, due to the lack of large-scale research, the importance of wildlife as a reservoir of AMR and zoonotic pathogens still seems to be insufficiently recognized.

To broaden our knowledge on diverse AMR and *Salmonella* transmission routes, the extensive surveillance of wildlife, especially with the use of sequencing techniques, is needed to reveal the zoonotic potential of bacteria originating from this reservoir. Whole-genome sequencing (WGS) of isolates derived from wild populations and representing diverse AMR profiles would allow for the determination of their phylogenetic relationships, identification of genes responsible for pathogenicity, characterization of plasmids contributing to horizontal transfer of virulence and AMR genes, and also to identify antimicrobial resistance determinants themselves. The application of this type of techniques could help to identify spreading routes for different pathogens and AMR, and will certainly help to answer the question about the direction of these transmissions. Is wildlife a vector that poses risk to other animal species, to the food production chain, and finally to humans, or whether the threat lies just on the other side [9,12]?

Transmission of pathogenic and resistant bacteria from wildlife to the bacterial gene pool in nature affects the ecosystem. This “microbial pollution” has an indisputable impact on different animal populations sharing the same habitats and thus may translate into the spread of pathogens and AMR determinants [13]. The analysis of animal populations in their natural habitats and presumably not exposed to anthropogenic impact gives the possibility to explore and reveal real hazards associated with wildlife [14]. In this context, studies of wild rodents offer great cognitive value as this abundant animal order is considered a reservoir of different bacteria of public health concern [15]. Rodents as one of the initial links in the trophic chain may serve as microbiological hazard transmitters for predators e.g., birds of prey, foxes, and others. Moreover, rodents can invade farmlands and thus they can pose a threat to farm animals or via livestock to the human population. 

Here, we applied methods adopted from official monitoring of slaughter animals to investigate both AMR and *Salmonella* occurrence in the wild rodent population in central and southeastern Poland. We tested a number of intestine samples derived from rodents coming from the natural habitats: meadows and forests. Our objective was to assess the occurrence of *Salmonella* and AMR of *E. coli* followed by the identification of AMR determinants and investigation of their epidemiological relationships and virulence potential. 

## 2. Results

### 2.1. Isolation Rates and Antimicrobial Resistance

**Figure 1:**
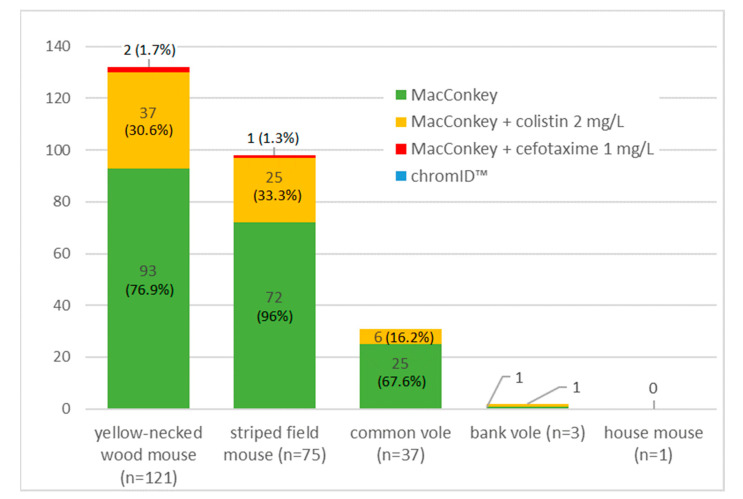
The number of *E. coli* strains by rodent species and isolation method.

From 237 intestine samples, no *Salmonella* spp. was isolated. A total of 263 *E. coli* were recovered. Indicator *E. coli* isolation rate scored 80.6% (*n* = 191). The recovery rate from supplemented MacConkey media was lower and equaled 1.3% (*n* = 3) for the medium with cefotaxime, and 29.1% (*n* = 69) for the medium with colistin added. No *E. coli* was isolated on media for carbapenem resistance screening. The number of isolated *E. coli* by rodent species and the selective medium used is presented in Figure 1.

The overall level of resistance among tested *E. coli* remained low. Minimal inhibitory concentration (MIC) values above the Epidemiological cut-off values (ECOFF) were found in 5 isolates (1.9%). Table 1 presents MIC values distribution of all tested *E. coli*. All of 191 commensal *E. coli* were pan-susceptible. Three strains recovered on cefotaxime supplemented medium showed MIC values for cefotaxime and ceftazidime just below the ECOFF (Figures S1–S3). Among *E. coli* isolated from the colistin-supplemented medium, one was resistant to tetracycline. Another four isolates were resistant to colistin (MIC range from 8 to 16 mg/L). All colistin-resistant isolates were recovered from females of the yellow-necked wood mouse. Animals were captured at different locations and on different dates in the summer 2016.

### 2.2. AMR Sequencing Results 

**Figure 2:**
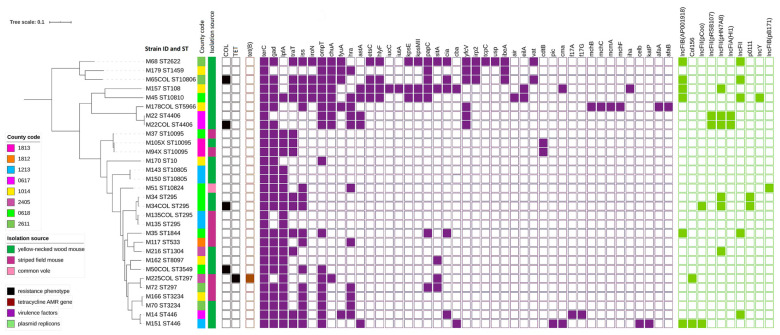
Phylogeny of *E. coli* strains isolated from wild rodents (sequence types, trapping site, source of isolation, and map of phenotypic resistance, resistance genes, virulence factors, and plasmid replicons).

Tetracycline resistance in one strain was due to *tet*(B) presence (Figure 2). In *E. coli* showing colistin, MIC > 2 mg/L WGS revealed neither the known plasmid-mediated *mcr* genes nor known mutations in *pmrA* and *pmrB* genes. Those strains exhibited several yet unknown point mutations in *pmrA* and *pmrB* but most of them were also found in colistin susceptible *E. coli* (Table S1). However, two colistin-resistant strains possessed single mutations that were not detected in any susceptible ones: *pmrB* p.L10R resulting in nucleotide change CTG into CGG and out of frame deletion *pmrB* p.L27_F31delinsL (Figure S4). Discrepancies between genotypic and phenotypic results were confirmed by repeated susceptibility testing (Figures S5–S8) followed by WGS retesting of the same culture. 

### 2.3. Plasmids Replicons

No evidence for plasmid occurrence was noted in 16 tested isolates. The remaining *E. coli* carried from 1 up to 3 plasmid incompatibility group replicons (Figure 2). Among them, replicons IncFII (pHN7A8) (*n* = 6), IncFIB (AP001918) (*n* = 6), IncFII (*n* = 5) were found the most often. All isolates with MIC values above the ECOFF, except one resistant to colistin, carried plasmid replicons. 

### 2.4. Virulence Genes

A diversity of virulence genes (*n* = 43) were noted in tested strains (Simpson diversity index D = 0.941). All tested strains carried virulence determinants (*n* ≥ 2). Seven *E. coli* contained more than 10 virulence genes from 11 up to 21. (Figure 2). Determinant *terC* was found in all tested *E. coli*. The other most prevalent genes were *gad* (*n* = 25), *lpfA* (*n* = 22), *ompT* (*n* = 17), and *iss* (*n* = 16). Shiga-toxin genes have not been identified in any of the tested strains.

### 2.5. Phylogenetic Analyses

Three isolates were excluded from the Multilocus sequence typing (MLST) summary since in three cases we sequenced 2 isolates derived from the same animal but different culture media. MLST results for all pairs were identical so we omitted one isolate from each pair. MLST of the remaining 27 *E. coli* revealed 20 sequence types (Simpson diversity index D = 0.977). Four ST types were noted for the first time: ST10805, ST10806, ST10810, ST10824 and added to the MLST database. Table S2 summarizes new STs with resulting loci and allele variants. Clonal group ST10095 was represented by 3 *E. coli* (Figure 2). Two strains derived from animals belonging to different rodent species (striped field mouse and yellow-necked wood mouse) but captured at the same site and date (13 September 2016). The third strain from this ST-type was derived from a striped field mouse trapped at a different site almost a month earlier (24 August 2016). Five clonal groups ST295, ST297, ST446, ST3234, ST10805 were represented by 2 strains each. ST10805 was noted in *E. coli* isolates coming from animals trapped at the same capture site and date. The remaining *E. coli* from those clonal groups were observed in animals originating from different trapping places. All resistant strains belonged to different STs. One colistin-resistant strain revealed new ST10806 (Figure 2). 

Within ST types represented by more than one strain (excluding isolates derived from the same animal), single nucleotide polymorphisms (SNPs) dissimilarities from 5 (between ST10805 isolates) up to 2594 (ST295) were noted. Among other ST types, the highest SNP difference 49,655 was noted between strains belonging to new ST types: 10810 (M45) and 10806 (M65 col).

## 3. Discussion

Identification of different wild animal populations as reservoirs of AMR determinants and *Salmonella* will aid in recognition and control of different AMR and pathogen transmission routes and thus to prevent threats and implications for human and animal health. Our study, covering a broad selection of wild rodents originating from distinct time and trapping sites, helped investigate the role of the tested subpopulation as an AMR and *Salmonella* vector. Several studies tested wild small mammals as sentinels of AMR and *Salmonella*, but yet, to the best of our knowledge, this study is the only one that applied WGS for characterization of obtained strains. Application of screening for cephalosporin-, carbapenem-, and colistin-resistance is definitely a strength of the current research.

All tested samples were free from *Salmonella* and this result is congruent with previous studies from the United Kingdom and Canada that regardless of the isolation method reported none or low level (1%) of *Salmonella* carriage in various rodent species including mice and voles derived from natural, landfill, and farm environments [16,17,18,19]. Those findings are contradictory to the research on commensal rodents that thrive in the vicinity of human settlements and can serve as vectors in pathogen transmission. High (49%) *Salmonella* prevalence in rats trapped on wet markets in Thailand was linked to probable close contact with other animal species and raw food, high temperature—in short: conditions conducive to the occurrence of *Salmonella* [20]. As shown in the study on city mice [21], rodents from urban areas also seem to be more prone to pathogen carriage.

A low level of *E. coli* resistance, including pan-susceptibility of the vast majority of strains, observed in the current study is congruent with other European studies. Similarly, infrequent AMR was presented by German research regarding small mammals (rodents and shrews) [22], where resistant commensal *E. coli* constituted 5.5% of all tested strains with beta-lactams-, aminoglycosides-, folate path inhibitors-, and tetracycline-resistances ranging from 0.5% up to 4%. An almost complete lack of resistance traits in enterobacteria derived from wild mammals *inter alia* bank voles was reported from Finland, where the only AMR found was towards cefuroxime and it was noted in few strains belonging to *Enterobacter agglomerans*, *Yersinia* spp., and *Serratia marcescens.* Among *E. coli* isolates, only one was resistant to streptomycin [23]. Other studies reported various carriage rates of AMR in wild rodents [18,19,24] and several pointed out that an undoubted factor affecting AMR prevalence in those animals was the proximity of livestock farms and human settlements, indicating an anthropogenic impact on AMR occurrence [18,19,22]. Canadian studies on small mammals revealed an association between AMR and origin of tested rodents and shrews—animals coming from natural areas were less likely to harbor resistant bacteria [18,19]. A higher resistance rate in *Enterobacterales* from wild rodents (i.e., 90% of coliforms being resistant to beta-lactams) was reported in northwest England [24]. The contrasts presented by both the current and above-referred studies show the complexity of the AMR phenomenon and indicate that factors affecting and contributing to AMR are not always easy to determine.

That also refers to our research and the presence of resistance to colistin, the last-resort treatment for humans [25]. An explanation for this finding is challenging. As already mentioned, the collected samples originated from rodents derived from their natural habitats and we may assume that animals had sporadic contact with anthropogenic impact and the overall AMR level would confirm these assumptions. A single study from England reported a high colistin AMR rate (over 30%) in *E. coli* from rodents, but in contrast to our research, most of the tested animals came from regions with clear anthropogenic influence e.g., farm or sewage treatment plant environment [26].

WGS of our colistin-resistant *E. coli* revealed neither plasmid-mediated *mcr* genes nor known mutations in *pmrA* and *pmrB* regions, however, we found several unknown mutations in *pmrA* and *pmrB.* In two cases, a mutational background of resistance mechanism may be excluded as the same mutations were found both in susceptible and colistin-resistant strains derived from the same animal. For now, we cannot disregard the emergence of new resistance mechanisms. Reduced susceptibility due to altered expression of here unidentified efflux pumps seems could also be an explanation [27]. Further analysis might elucidate the exact mechanism of resistance. The molecular background of colistin resistance has not been reported or fully investigated in bacteria from wild mammals, although the presence of the *mcr-1* gene was excluded also in previous studies [26,28]. 

As no resistance breakpoint for azithromycin in *E. coli* has been adopted so far, we decided to sequence a few strains with MIC of 8–16 mg/L to investigate the presence of presumptive AMR determinants. The observed lack of acquired genes and specific mutations determining resistance for macrolides is congruent with previous reports on missing mechanisms of resistance in the vast majority of *E. coli* with azithromycin MIC ≤ 32 mg/L [29]. The same research revealed that MICs higher than 32 mg/L were associated with the presence of *mph(A)*. Neither this gene nor high MICs were found in our study. 

The overall level of AMR noted here might be associated with a low abundance of plasmid replicons. Considering the role of plasmids in AMR dissemination, it needs to be highlighted that more than half of the analyzed *E. coli* were plasmid-free. Anyhow, several virulence factors were found in tested *E. coli* and, among strains, with the highest number of virulence genes (above 10), the majority carried up to 3 plasmid replicons. Evidence of replicon IncFII (pCoo) would indicate the presence of pCoo virulence plasmid associated with enterotoxigenic *E. coli* (ETEC) [30,31]. Identified replicon Col156 was previously reported in clinical isolates of *Shigella sonnei* and *flexneri* but also multidrug-resistant clinical *E. coli* [32,33,34].

We presume that the low level of AMR in our *E. coli* might also be related to the sizeable amount of virulence determinants found. Particularly in the absence of a selective pressure, this would rationalize the compensation of fitness costs for bacterial cells [35].

It is worth to note that among tested *E. coli*, three possessed the *ibeA* gene encoding invasin of brain endothelial cells that occurs in *E. coli* associated with meningitis during the neonatal period [36,37]. The gene encoding the cytolethal distending toxin (CDT) reported in various clinical isolates of *E. coli* was noted as well [38,39]. Moreover, one strain harbored the *air* gene encoding the enteroaggregative immunoglobulin repeat protein and also the *eilA* gene, which activates the surface protein Air [40]. Additionally, we found enteroaggregative heat-stable toxin 1 *astA* gene in those strains. The EAST1 toxin encoded by that gene has been reported in clinically relevant types of enteric *E. coli* [41]. The *vat* and *pic* determinants (serine protease autotransporters of Enterobacteriaceae—SPATE) described here are commonly found in the pathotype responsible for acute and persistent diarrhea—enteroaggregative *E. coli* (EAEC). However, due to the absence of *aggR* regulon, current *E. coli* could be recognized rather as atypical EAEC [42,43]. We also found a strain possessing the gene encoding adhesin F17 fimbriae that was described in pathogenic *E. coli* strains isolated from diarrhoeic calves [44]. It should be underlined that the most prevalent genes we noted (*lpfA* and *iss*) are widely distributed among *E. coli* pathotypes [43,45].

Our attention was drawn to the genomic heterogeneity of the sequenced strains. Most of them represented unique sequence types and some were revealed for the first time. Further, certain STs represented by several *E. coli* isolated from animals captured at different locations and time slots, might suggest common autochthonous rodent-specific gut microorganisms. Based on the same STs found in isolates from different rodent species and similar location, we may assume association resulting from the common habitat of the animals. 

Worth emphasizing is the fact that one of the tested strains belonged to ST10 lineage reported in human clinical cases with extraintestinal pathogenic *E. coli* (ExPEC) including uropathogenic *E. coli* (UPEC) [46,47,48], yet our strain was pan-susceptible and possessed only three virulence factors. 

Although a limited number of strains were analyzed in that context, the results highlighted genetic diversity and the structure of *E. coli* derived from wild rodents. Additionally, our study revealed their potential for virulence factors transmission and enhanced capacity for intestinal colonization, indicating presumed pathogenicity of tested strains.

## 4. Materials and Methods 

### 4.1. Sample Collection

**Figure 3:**
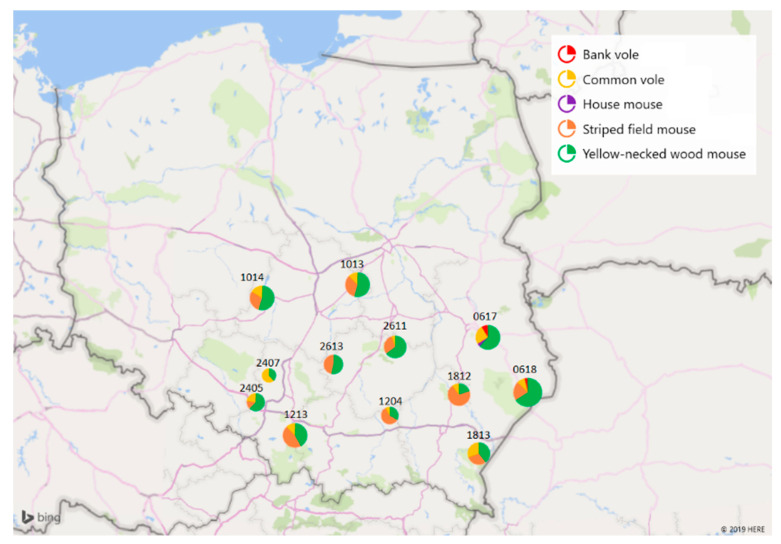
Distribution of animals over the territory of Poland. The numbers correspond to the territorial numbers of local administrative units (counties). Visualization with Microsoft 3D Maps for Excel (2016).

A total of 237 intestine samples recovered from five rodent species: yellow-necked wood mouse (*Apodemus flavicollis*, *n* = 121), striped field mouse (*Apodemus agrarius*, *n* = 75), common vole (*Microtus arvalis*, *n* = 37), bank vole (*Myodes glareolus*, *n* = 3), and house mouse (*Mus musculus*, *n* = 1) were collected by the National Reference Laboratory for *Salmonella* and antimicrobial resistance (NRL) at the National Veterinary Research Institute (NVRI). Animals were caught between 2016 and 2017 in forests and meadows of central, eastern, and southeastern Poland within the National Science Centre (NCN) grant: “Environment of free-living and companion animals—the potential source of zoonotic Leptospira strains” (UMO-2013/09/B/NZ7/02563). Distribution of animals trapping sites over the territory of Poland is presented in Figure 3. All procedures for wild animal capture and handling were approved by the Local Ethics Committee for Animal Experimentation in Lublin (Resolution No. 30/2016). Details on the trapping technique were previously described [49]. Immediately after being caught, the animals were transported to NVRI and euthanized on the same day. Necropsies and intestinal sampling were performed under aseptic conditions under laminar air flow. Instantly after collection, the intestines were frozen and stored at <−80 °C until processing.

### 4.2. Bacterial Isolation and Identification 

Thawing of samples was done only once, just before testing. All samples were first cultured in buffered peptone water for 18 ± 3 h at 37 °C. After pre-incubation, the samples were simultaneously tested for *Salmonella* spp. according to ISO 6579-1:2017-04 standard and *E. coli* isolation methods used in official AMR monitoring (652/2013/EC Commission Implementing Decision) [50]. For isolation of commensal, cephalosporin-, and carbapenem-resistant *E. coli*, each sample was simultaneously streaked on, respectively, MacConkey agar (Oxoid, Hampshire, UK), MacConkey agar supplemented with cefotaxime (1 mg/L, Oxoid, Hampshire, UK), chromID™ CARBA, and chromID™ OXA-48 agar (bioMérieux, Marcy l’Etoile, France). Additionally, samples were streaked on MacConkey supplemented with colistin (2 mg/L, Oxoid, Hampshire, UK) for detection of colistin-resistant *E. coli*. Presumed *E. coli* were identified with polymerase chain reaction (PCR) targeting the universal shock protein A gene (*uspA*) according to a previously described protocol [51].

### 4.3. Antimicrobial Resistance Testing

Antimicrobial susceptibility testing of all confirmed bacterial isolates was performed with the broth dilution method (Sensititre EUVSEC plates; TREK Diagnostic Systems, Thermo Fisher Scientific, Waltham, MA, USA). Epidemiological cut-off values (ECOFFs) according to the European Committee on Antimicrobial Susceptibility Testing (EUCAST) for minimal inhibitory concentration (MICs) obtained for 14 compounds representing 9 antimicrobial classes: beta-lactams, quinolones, phenicols, aminoglycosides, folate-path inhibitors, tetracyclines, polymyxins, macrolides, and glycylcyclines were used as interpretation criteria as formerly described [52]. For each substance with MIC above the cut-off, the isolate was regarded as microbiologically resistant (non-wild type, NWT).

### 4.4. Whole-Genome Sequencing 

A subset (*n* = 30) of bacterial isolates were selected for whole genome sequencing. WGS analysis covered five resistant strains (four colistin- and one tetracycline-resistant), 10 strains with MICs for azithromycin 8–16 mg/L, and 15 pan-susceptible *E. coli*. DNA was extracted with the genomic mini kit according to the manufacturer’s instructions (A&A Biotechnology, Gdynia, Poland) and measured for yield and purity check (NanoDrop™ One, Thermo Fisher Scientific, Waltham, MA, USA). DNA libraries were prepared with the library preparation kit (Illumina, Inc., San Diego, CA, USA) and sequenced with the MiSeq platform (Illumina, Inc., San Diego, CA, USA), using 2 × 300 paired-end sequencing per flowcell resulting in ~0.4 Gbps per sample.

### 4.5. Bioinformatic Processing and Data Analysis

The quality of raw reads was checked with FastQC 0.11.5 and next trimmed by Trimmomatic 0.36 [53]. Corrected reads were assembled de novo by SPAdes 3.9.0. [54]. The sequences were deposited at the European Nucleotide Archive (ENA) under accession number: PRJEB39482.

In silico bioinformatic tools by the Center for Genomic Epidemiology (CGE) were applied for analyses of assembled sequences: ResFinder software: 3.2 (31 March 2020) with ResFinder database (8 April 2020) and PointFinder software: 3.1.0 (27 February 2019) with its database: (2 July 2019) for identification of resistance determinants [55], MLST 2.0 (Multi-Locus Sequence Typing Software version: 2.0.4 (8 May 2019) for identification of multilocus sequence type (MLST, ST) [56] with database version: 2.0.0 (4 May 2020), VirulenceFinder 2.0 Software version: (21 May 2020) and its database version: (29 May 2020) [57] for characterization of virulence factors. In the case of ResFinder, PointFinder and VirulenceFinder selected %ID threshold was 90% and selected minimum length 60%. Plasmid identification was conducted using PlasmidFinder software version: 2.0.1 (7 February 2020) and database (2 April 2020) with threshold 95% and selected minimum length 60% [58].

Isolates with unknown ST were submitted to EnteroBase v1.1.2 (https://enterobase.warwick.ac.uk/) and have assigned new sequence type using Achtman 7 Gene MLST algorithm [59].

Phylogeny tree was prepared with CSI Phylogeny 1.4 (Call SNPs & Infer Phylogeny) CGE with input parameters—minimum depth at SNP positions: 10, relative depth at SNP positions: 10, minimum distance between SNPs (prune): 10, minimum SNP quality: 30, minimum read mapping quality: 25, minimum Z-score: 1.96 [60]. The genome of a strain belonging to the most represented ST type was chosen as reference (M37). 

For visualization, iTOL v5, an online tool, was applied [61]. The variability of noted MLST and virulence genes was measured with Simpson’s diversity index [62]. 

## 5. Conclusions

Our study did not reveal any public health relevance of the sampled wild rodent population in *Salmonella* spread. Moreover, the low abundance of resistance in *E. coli* (both on phenotype and genotype level) indicated a limited role of that group of animals in AMR dissemination on the tested territory. The study broadened our knowledge of phylogenetic diversity and structure of *E. coli* population in wild rodents and suggests that it may serve as a reservoir of several virulence factors. 

## Figures and Tables

**Table 1:** Distribution of minimal inhibitory concentration of isolated *E. coli* (N = 263).

Antimicrobial Name and Abbreviation	NWT		Minimal Inhibitory Concentration Value (mg/L)																		
	*n*	%	≤0.008	0.016	0.032	0.064	0.125	0.25	0.5	1	2	4	8	16	32	64	128	256	512	1024	>1024
Ampicillin (AMP)	0	0.0%							0	17	177	69	0	0	0	0	0				
Ceftazidime (TAZ)	0	0.0%						0	263	0	0	0	0	0							
Cefotaxime (FOT)	0	0.0%					0	263	0	0	0	0	0								
Meropenem (MERO)	0	0.0%		0	261	2	0	0	0	0	0	0	0	0	0						
Gentamicin (GEN)	0	0.0%						0	116	127	20	0	0	0	0	0					
Nalidixic acid (NAL)	0	0.0%									0	262	1	0	0	0	0	0			
Ciprofloxacin (CIP)	0	0.0%	0	232	30	1	0	0	0	0	0	0	0	0							
Sulfamethoxazole (SMX)	0	0.0%										0	74	65	56	68	0	0	0	0	0
Trimethoprim (TMP)	0	0.0%					0	233	28	2	0	0	0	0	0	0					
Colistin (COL)	4	1.5%							0	259	0	0	1	3	0	0	0				
Azithromycin (AZI)		NI								0	127	69	61	6	0	0	0				
Chloramphenicol (CHL)	0	0.0%										0	263	0	0	0	0	0			
Tetracycline (TET)	1	0.4%								0	262	0	0	0	0	1	0				
Tigecycline (TIGECY)	0	0.0%					0	259	4	0	0	0	0	0							

Red vertical lines indicate EUCAST epidemiological cutoff values applied as interpretative criteria. NWT—non-wild type, defines microbiologically resistant isolates with MIC value higher than the epidemiological cutoff value. NI—no interpretation criteria available.

## Supplementary Materials

The following are available online at https://www.mdpi.com/2076-0817/9/9/771/s1, Figure S1. MIC results of strain M93X derived on cefotaxim supplemented medium, Figure S2. MIC results of strain M94X derived on cefotaxim supplemented medium, Figure S3. MIC results of strain M105X derived on cefotaxim supplemented medium, Figure S4: Mapping of reads from strain M50 col to *pmrB* reference from PoitFinder database confirming deletion in *pmrB* p.L27_F31delinsL, Figure S5: MIC results of *E. coli* strain resistant to colistin-M22 col, Figure S6: MIC results of *E. coli* strain resistant to colistin-M34 col, Figure S7: MIC results of *E. coli* strain resistant to colistin-M50 col, Figure S8: MIC results of *E. coli* strain resistant to colistin-M65 col, Table S1: Chromosomal point mutations in *pmrA* and *pmrB* noted in colistin-resistant strains, Table S2: Loci and allele variants resulting in novel *E. coli* sequence types.
