# Supplementary material for: Salmonella and Antimicrobial Resistance in Wild Rodents—True or False Threat?
Source: Pathogens. 2020 Sep 21;9(9):771. doi: 10.3390/pathogens9090771 (PMC7559071; doi:10.3390/pathogens9090771)
Supplement: Supplementary file 1 [file pathogens-09-00771-s001.zip › supplementary_filesv6/Table S1. Chromosomal point mutations in pmrA and pmrB noted in colistin resistant strains.pdf]

Table S1. Chromosomal point mutations in *pmrA* and *pmrB* noted in colistin resistant strains.

Isolate & MIC	Mutation	Nucleotide change	Amino acid change
M22col MIC 16 M22 MIC <=1	pmrA p.G53S	GGG -> AGC	G -> S
	pmrA p.N222K	AAC -> AAA	N -> K
	pmrB p.H2R	CAT -> CGT	H -> R
	pmrB p.D283G	GAC -> GGC	D -> G
	pmrB p.A360V	GCA -> GTA	A -> V
M34col MIC 16 M34 MIC<=1	pmrB p.A159V	GCG -> GTT	A -> V
	pmrB p.D283G	GAC -> GGC	D -> G
	pmrB p.Y358N	TAC -> AAC	Y -> N
M50 col MIC 8	pmrB p.L27_F31delinsL	CTGATCAGCGTCTTC -> CT------------C	LISVF -> L
	pmrB p.D283G	GAC -> GGC	D -> G
	pmrB p.Y358N	TAC -> AAC	Y -> N
M65col MIC 16	pmrA p.T31S	ACA -> AGC	T -> S
	pmrA p.I128N	ATT -> AAC	I -> N
	pmrA p.G144S	GGC -> AGC	G -> S
	pmrB p.H2R	CAT -> CGT	H -> R
	pmrB p.L10R	CTG -> CGG	L -> R
	pmrB p.E123D	GAA -> GAT	E -> D
	pmrB p.D283G	GAC -> GGC	D -> G
	pmrB p.V351I	GTA -> ATA	V -> I
