# Supplementary material for: Salmonella and Antimicrobial Resistance in Wild Rodents—True or False Threat?
Source: Pathogens. 2020 Sep 21;9(9):771. doi: 10.3390/pathogens9090771 (PMC7559071; doi:10.3390/pathogens9090771)
Supplement: Supplementary file 1 [file pathogens-09-00771-s001.zip › supplementary_filesv6/Table S2. Loci and allele variants resulting in novel E. coli sequence types.pdf]

Table S2. Loci and allele variants resulting in novel *E. coli* sequence types.

**MLST**	**allele variants**						
	adk	***fumC***	***gyrB***	***icd***	***mdh***	***purA***	***recA***
ST10805	6	4	782	1267	11	239	7
ST10806	76	568	247	36	17	14	28
ST10810	18	22	16	23	5	138	4
ST10824	6	19	14	16	11	12	156
