# Supplementary figures and images for: Salmonella and Antimicrobial Resistance in Wild Rodents—True or False Threat?
Source: Pathogens. 2020 Sep 21;9(9):771. doi: 10.3390/pathogens9090771 (PMC7559071; doi:10.3390/pathogens9090771)

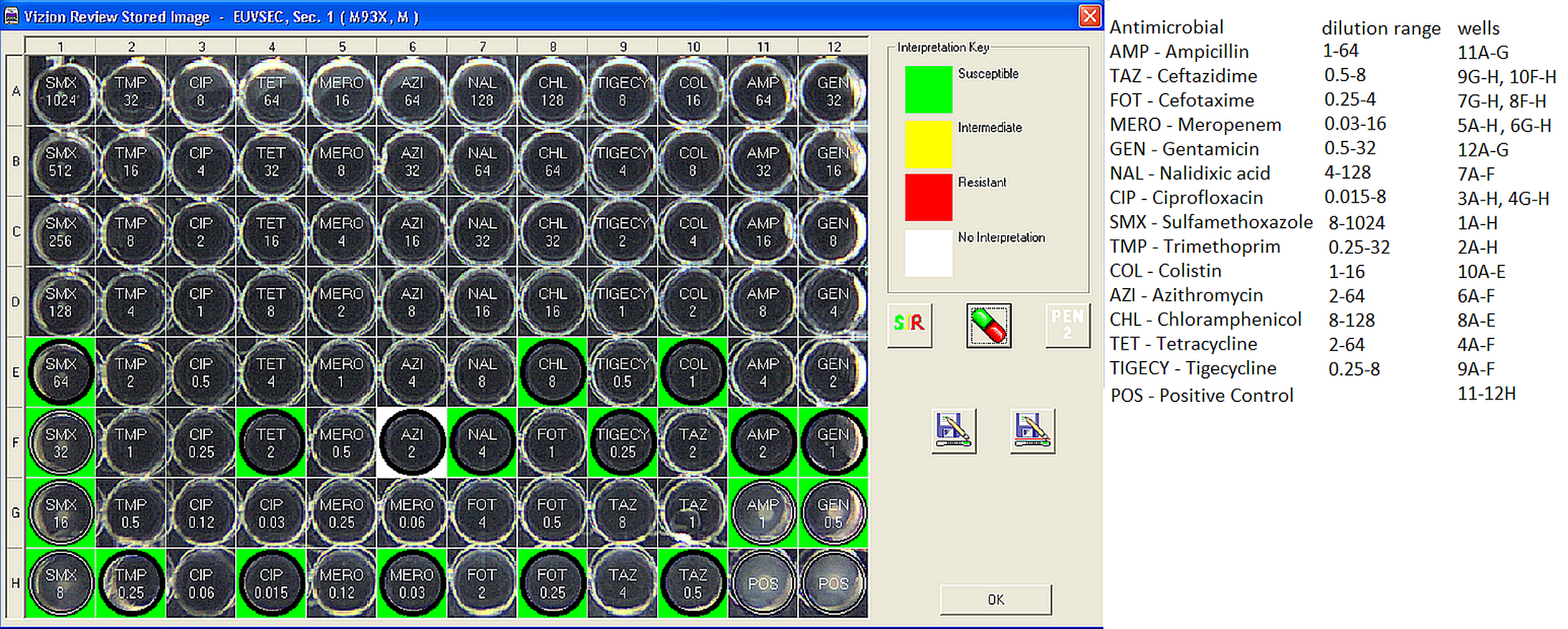

Supplement: Supplementary file 1 [file pathogens-09-00771-s001.zip › supplementary_filesv6/Figure S1. MIC results of strain M93X derived on cefotaxim supplemented medium.tif]

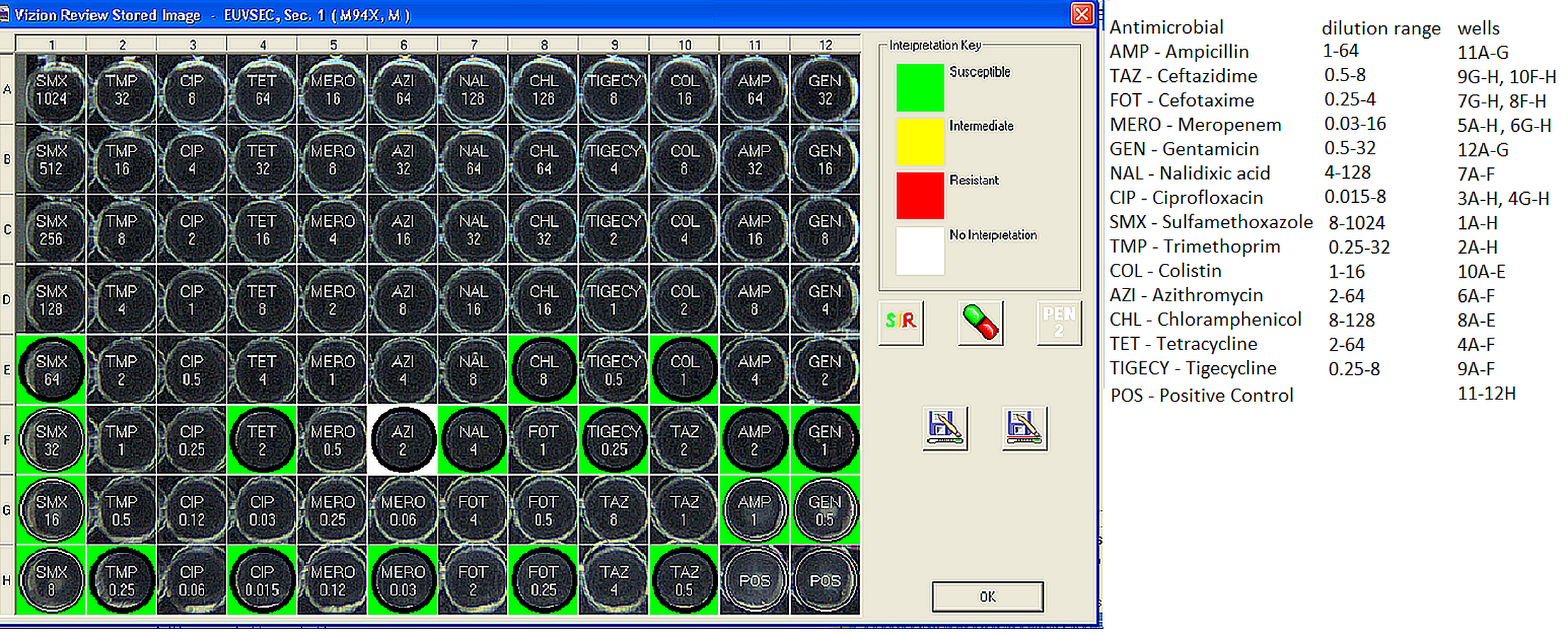

Supplement: Supplementary file 1 [file pathogens-09-00771-s001.zip › supplementary_filesv6/Figure S2. MIC results of strain M94X derived on cefotaxim supplemented medium.tif]

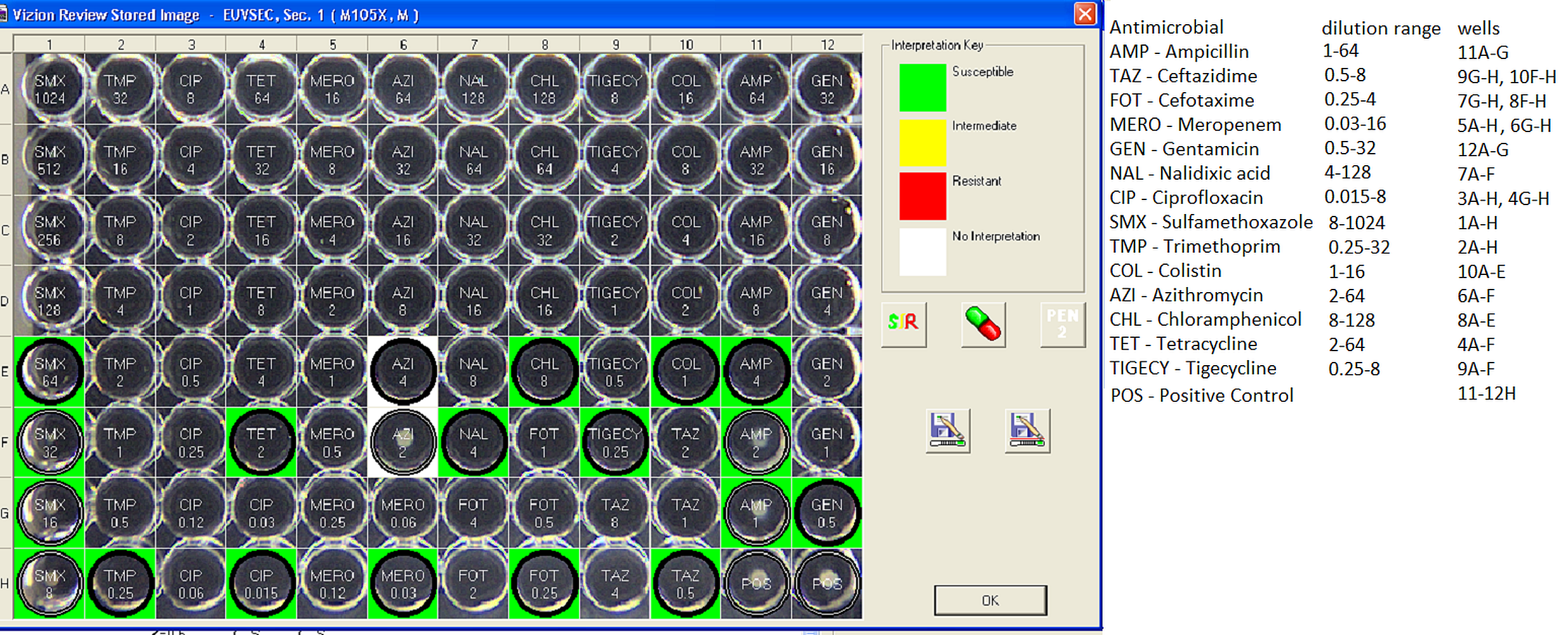

Supplement: Supplementary file 1 [file pathogens-09-00771-s001.zip › supplementary_filesv6/Figure S3. MIC results of strain M105X derived on cefotaxim supplemented medium.tif]

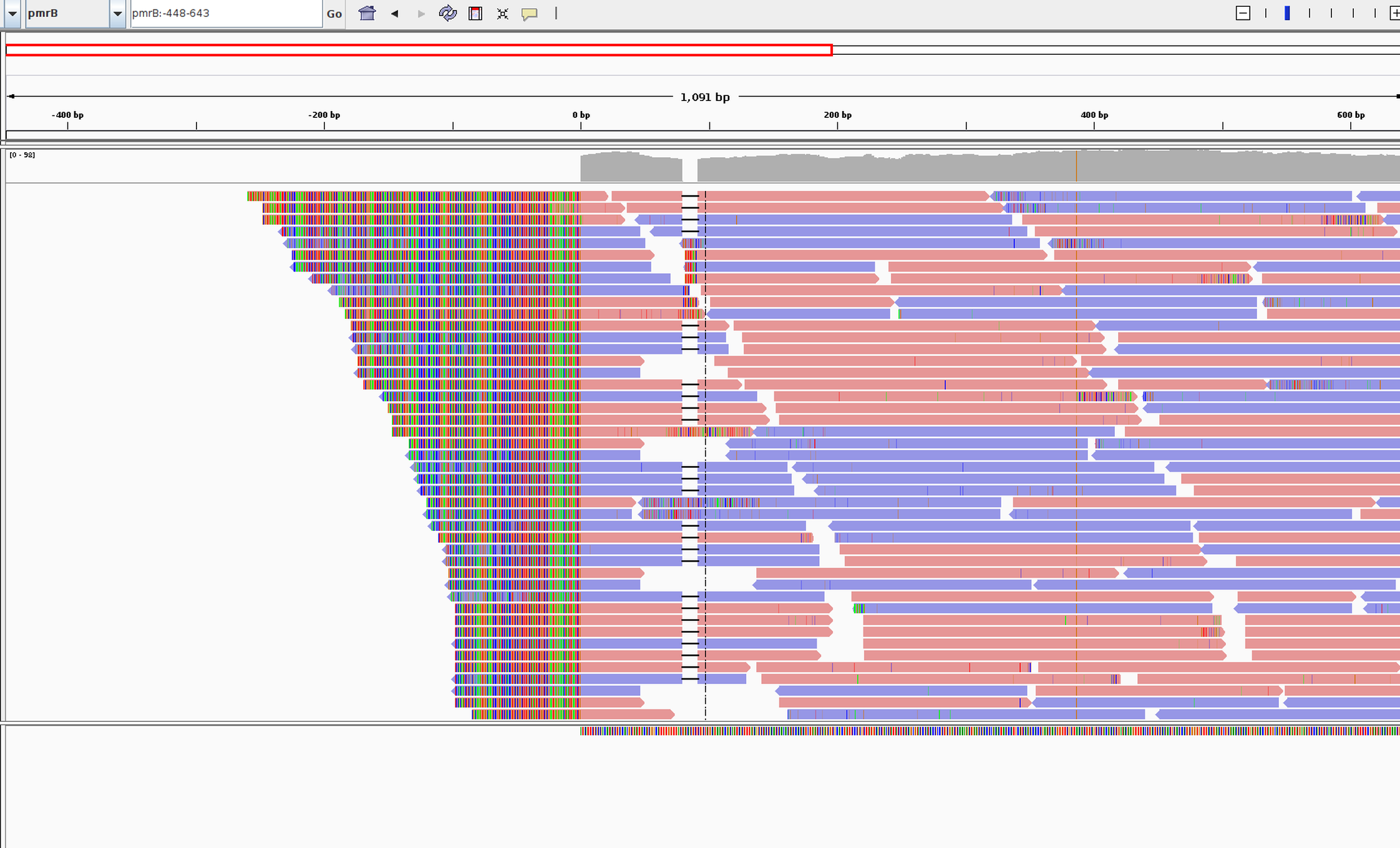

Supplement: Supplementary file 1 [file pathogens-09-00771-s001.zip › supplementary_filesv6/Figure S4. Mapping of reads from strain M50 col to pmrB reference from PoitFinder database confirming deletion in pmrB p.L27_F31delinsL.tif]

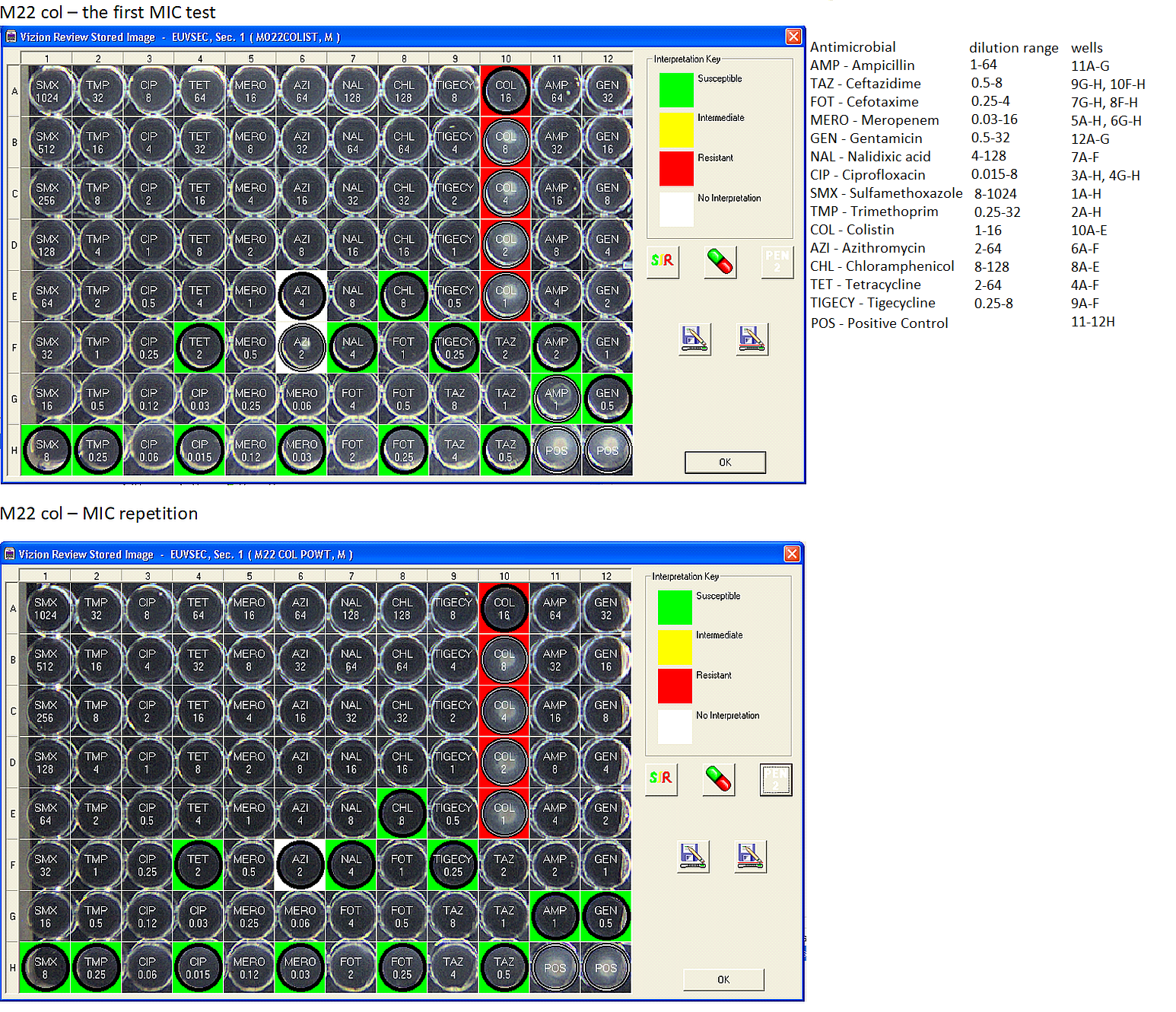

Supplement: Supplementary file 1 [file pathogens-09-00771-s001.zip › supplementary_filesv6/Figure S5 . MIC results of E. coli strains resistant to colistin M22 col.tif]

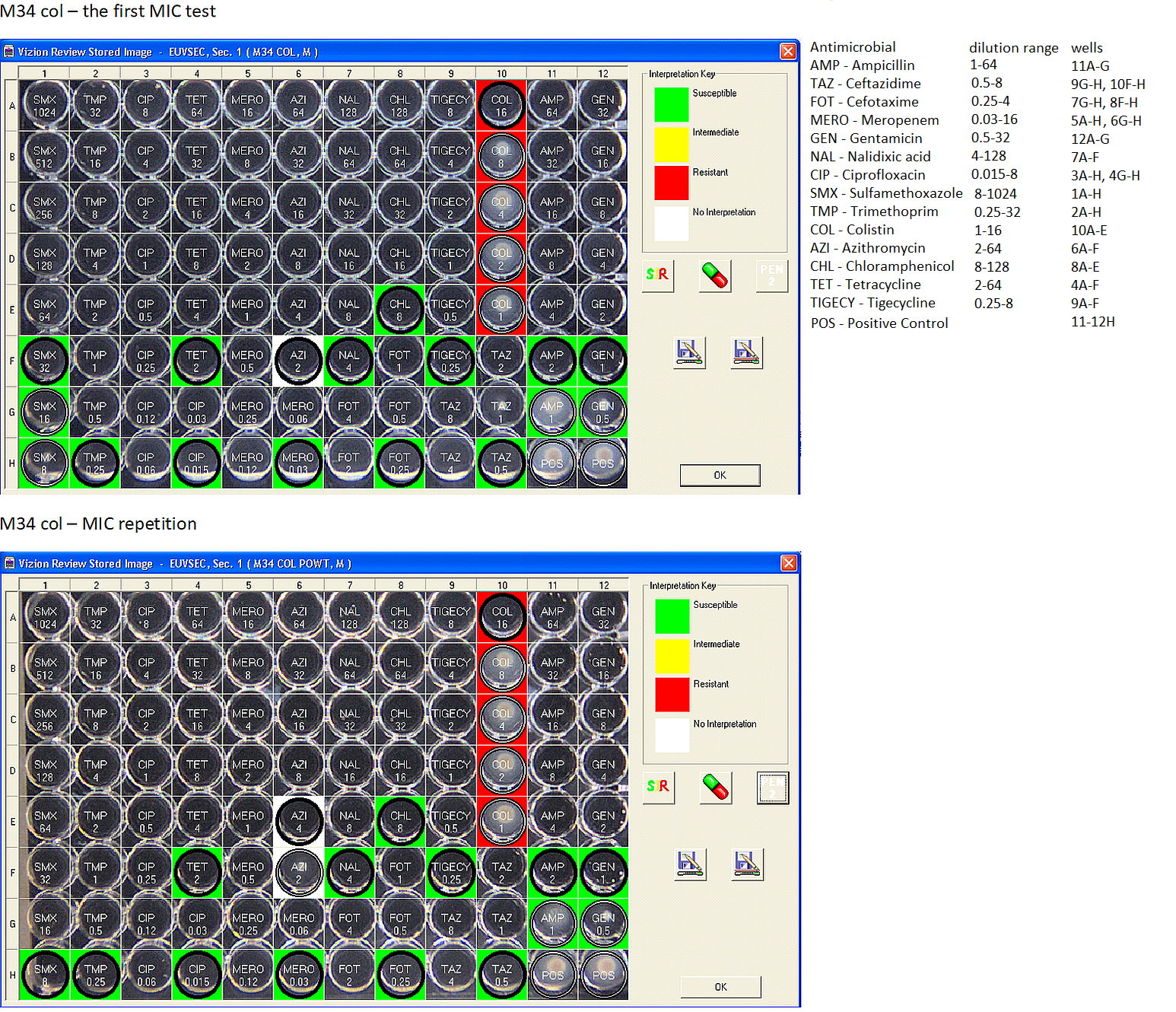

Supplement: Supplementary file 1 [file pathogens-09-00771-s001.zip › supplementary_filesv6/Figure S6 . MIC results of E. coli strains resistant to colistin M34 col.tif]

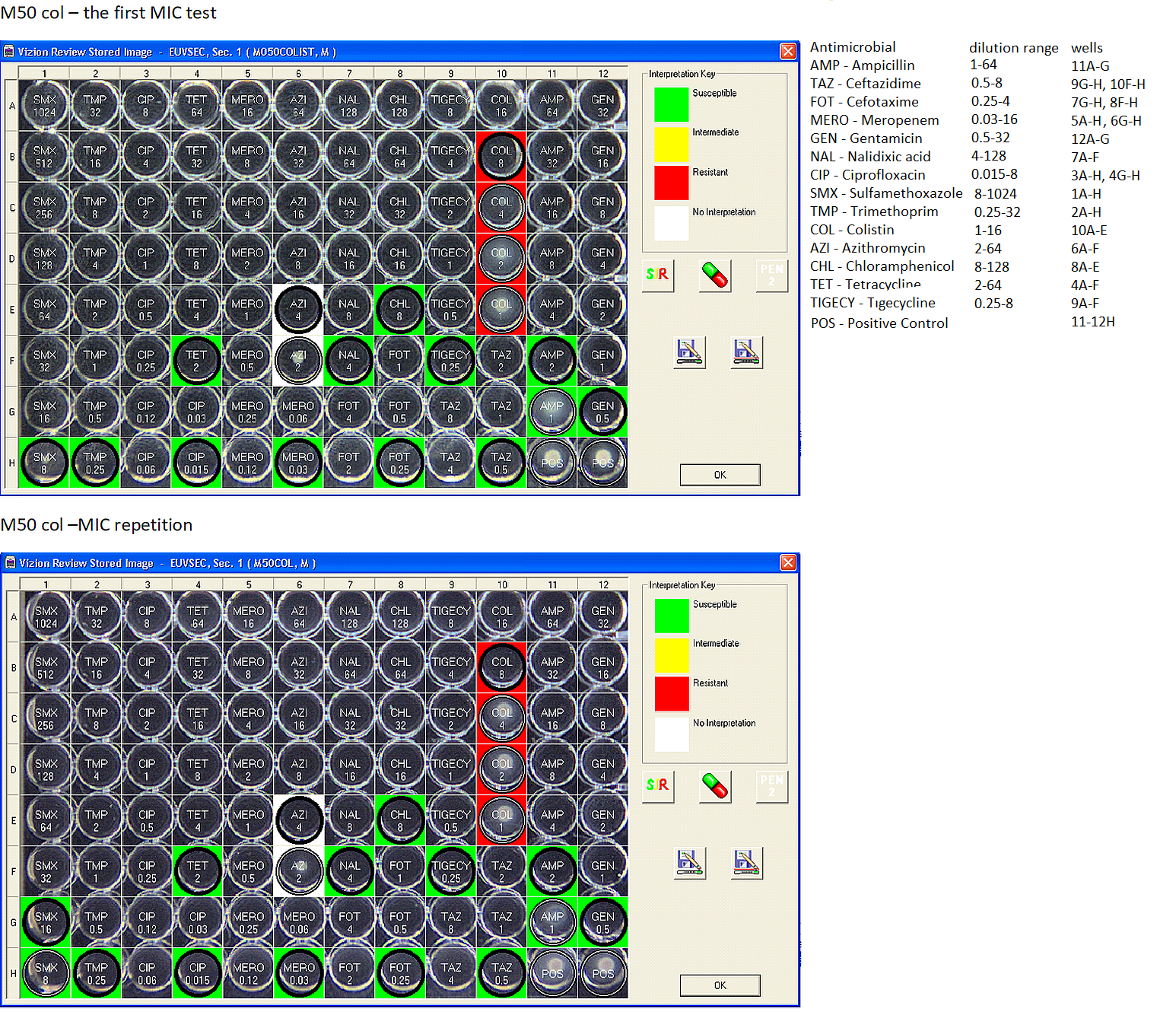

Supplement: Supplementary file 1 [file pathogens-09-00771-s001.zip › supplementary_filesv6/Figure S7 . MIC results of E. coli strains resistant to colistin M50 col.tif]

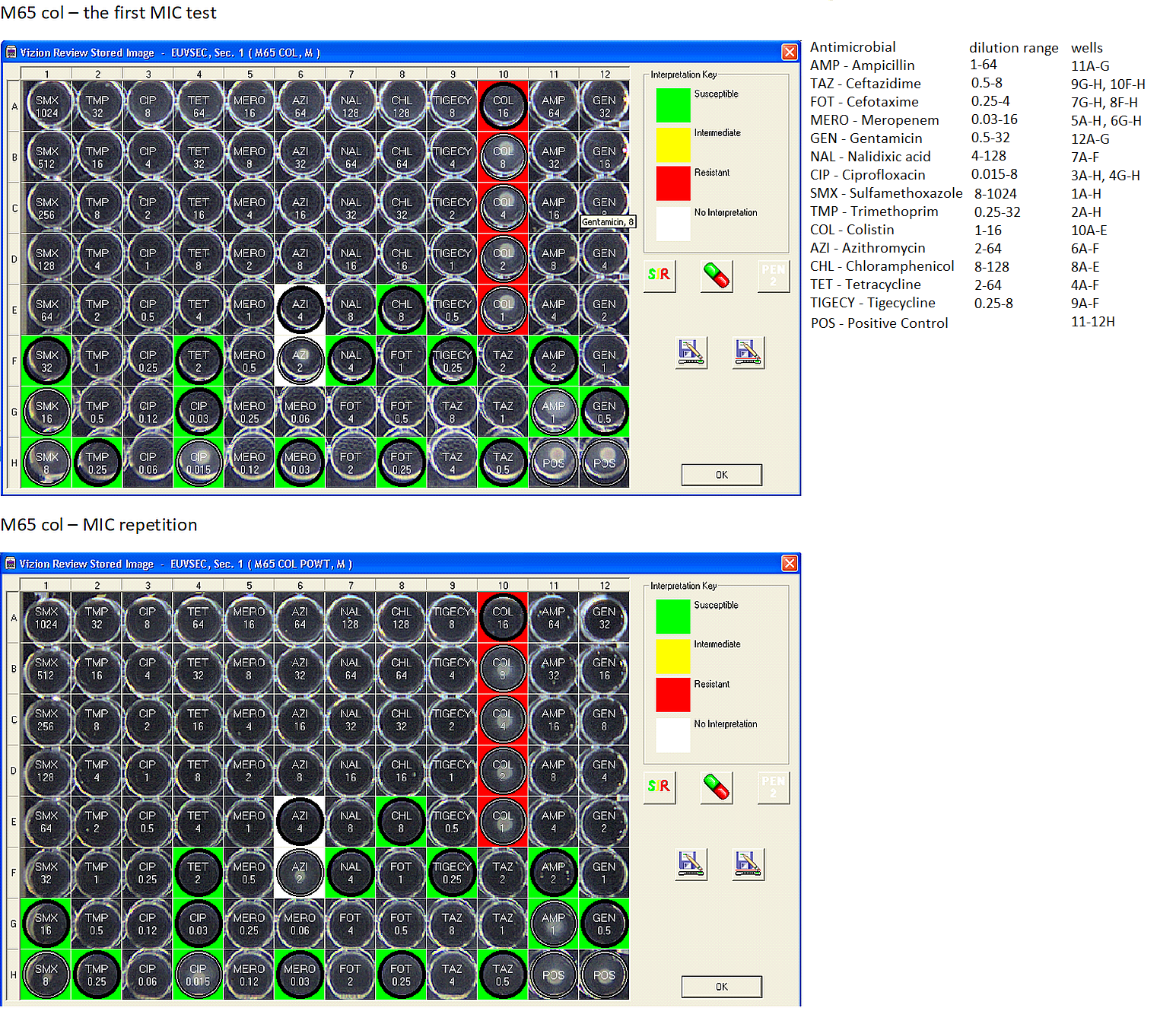

Supplement: Supplementary file 1 [file pathogens-09-00771-s001.zip › supplementary_filesv6/Figure S8 . MIC results of E. coli strains resistant to colistin M65 col.tif]
